# Supplementary material for: Modeling glioblastoma heterogeneity as a dynamic network of cell states
Source: Mol Syst Biol. 2021 Sep 16;17(9):e10105. doi: 10.15252/msb.202010105 (PMC8444284; doi:10.15252/msb.202010105)
Supplement: Supplementary file 5 — Source Data for Figure 3 [file MSB-17-e10105-s001.zip › Figure3A_sourcedata/GSEA_3065/hallmarks_state5.GseaPreranked.1623416302988/HALLMARK_P53_PATHWAY.html]

Details for gene set HALLMARK\_P53\_PATHWAY[GSEA]

|  || Dataset | state5 |
| Phenotype | NoPhenotypeAvailable |
| Upregulated in class | na\_neg |
| GeneSet | HALLMARK\_P53\_PATHWAY |
| Enrichment Score (ES) | -0.33162546 |
| Normalized Enrichment Score (NES) | -1.1855215 |
| Nominal p-value | 0.17418547 |
| FDR q-value | 0.40986216 |
| FWER p-Value | 1.0 |
Table: GSEA Results Summary

  

Fig 1: Enrichment plot: HALLMARK\_P53\_PATHWAY      
 Profile of the Running ES Score & Positions of GeneSet Members on the Rank Ordered List

  

| PROBE | GENE SYMBOL | GENE\_TITLE | RANK IN GENE LIST | RANK METRIC SCORE | RUNNING ES | CORE ENRICHMENT || 1 | APP |  |  | 38 | 0.712 | 0.0265 | No |
| 2 | CD81 |  |  | 159 | 0.475 | 0.0335 | No |
| 3 | SLC3A2 |  |  | 222 | 0.424 | 0.0448 | No |
| 4 | NOTCH1 |  |  | 348 | 0.361 | 0.0463 | No |
| 5 | CTSD |  |  | 388 | 0.347 | 0.0570 | No |
| 6 | SEC61A1 |  |  | 406 | 0.338 | 0.0696 | No |
| 7 | RAD9A |  |  | 418 | 0.335 | 0.0829 | No |
| 8 | CTSF |  |  | 438 | 0.329 | 0.0949 | No |
| 9 | TM7SF3 |  |  | 508 | 0.308 | 0.1005 | No |
| 10 | F2R |  |  | 568 | 0.293 | 0.1065 | No |
| 11 | PLXNB2 |  |  | 577 | 0.291 | 0.1181 | No |
| 12 | DDB2 |  |  | 833 | 0.235 | 0.0995 | No |
| 13 | PTPRE |  |  | 893 | 0.227 | 0.1026 | No |
| 14 | TAP1 |  |  | 1077 | 0.202 | 0.0907 | No |
| 15 | POM121 |  |  | 1285 | 0.173 | 0.0748 | No |
| 16 | FOS |  |  | 1412 | 0.161 | 0.0675 | No |
| 17 | ABCC5 |  |  | 1456 | 0.157 | 0.0694 | No |
| 18 | SDC1 |  |  | 1464 | 0.156 | 0.0754 | No |
| 19 | FUCA1 |  |  | 1611 | 0.141 | 0.0650 | No |
| 20 | ANKRA2 |  |  | 1613 | 0.141 | 0.0709 | No |
| 21 | IP6K2 |  |  | 1621 | 0.140 | 0.0762 | No |
| 22 | ITGB4 |  |  | 1681 | 0.135 | 0.0754 | No |
| 23 | EPHX1 |  |  | 1903 | 0.118 | 0.0555 | No |
| 24 | PIDD1 |  |  | 1948 | 0.115 | 0.0555 | No |
| 25 | PERP |  |  | 1950 | 0.115 | 0.0604 | No |
| 26 | PRMT2 |  |  | 2020 | 0.110 | 0.0573 | No |
| 27 | CD82 |  |  | 2145 | 0.102 | 0.0478 | No |
| 28 | BTG2 |  |  | 2174 | 0.100 | 0.0489 | No |
| 29 | AEN |  |  | 2225 | 0.096 | 0.0474 | No |
| 30 | BAIAP2 |  |  | 2289 | 0.092 | 0.0443 | No |
| 31 | PVT1 |  |  | 2345 | 0.089 | 0.0419 | No |
| 32 | CGRRF1 |  |  | 2376 | 0.087 | 0.0423 | No |
| 33 | STEAP3 |  |  | 2544 | 0.077 | 0.0268 | No |
| 34 | SLC7A11 |  |  | 2577 | 0.075 | 0.0264 | No |
| 35 | RAB40C |  |  | 2609 | 0.073 | 0.0261 | No |
| 36 | BLCAP |  |  | 2702 | 0.068 | 0.0186 | No |
| 37 | HDAC3 |  |  | 2812 | 0.062 | 0.0090 | No |
| 38 | CCNK |  |  | 2878 | 0.059 | 0.0042 | No |
| 39 | PLK3 |  |  | 2909 | 0.057 | 0.0033 | No |
| 40 | DRAM1 |  |  | 2940 | 0.056 | 0.0023 | No |
| 41 | PDGFA |  |  | 2967 | 0.054 | 0.0017 | No |
| 42 | EPHA2 |  |  | 3013 | 0.051 | -0.0012 | No |
| 43 | ABAT |  |  | 3020 | 0.051 | 0.0003 | No |
| 44 | DNTTIP2 |  |  | 3040 | 0.050 | 0.0003 | No |
| 45 | TM4SF1 |  |  | 3045 | 0.050 | 0.0020 | No |
| 46 | SLC35D1 |  |  | 3070 | 0.048 | 0.0014 | No |
| 47 | CCP110 |  |  | 3202 | 0.041 | -0.0116 | No |
| 48 | IER5 |  |  | 3319 | 0.035 | -0.0232 | No |
| 49 | HSPA4L |  |  | 3471 | 0.028 | -0.0390 | No |
| 50 | TGFB1 |  |  | 3503 | 0.027 | -0.0414 | No |
| 51 | SP1 |  |  | 3520 | 0.026 | -0.0420 | No |
| 52 | STOM |  |  | 3625 | 0.023 | -0.0528 | No |
| 53 | WRAP73 |  |  | 3690 | 0.020 | -0.0591 | No |
| 54 | TRAFD1 |  |  | 4009 | 0.007 | -0.0948 | No |
| 55 | MKNK2 |  |  | 4083 | 0.004 | -0.1029 | No |
| 56 | ATF3 |  |  | 4225 | -0.002 | -0.1187 | No |
| 57 | CDKN2AIP |  |  | 4245 | -0.003 | -0.1207 | No |
| 58 | NOL8 |  |  | 4248 | -0.003 | -0.1208 | No |
| 59 | SESN1 |  |  | 4251 | -0.003 | -0.1209 | No |
| 60 | VWA5A |  |  | 4356 | -0.008 | -0.1323 | No |
| 61 | XPC |  |  | 4377 | -0.009 | -0.1341 | No |
| 62 | RXRA |  |  | 4441 | -0.012 | -0.1407 | No |
| 63 | CCND2 |  |  | 4539 | -0.016 | -0.1510 | No |
| 64 | PITPNC1 |  |  | 4565 | -0.017 | -0.1531 | No |
| 65 | CSRNP2 |  |  | 4618 | -0.019 | -0.1582 | No |
| 66 | KIF13B |  |  | 4678 | -0.021 | -0.1639 | No |
| 67 | BMP2 |  |  | 4770 | -0.025 | -0.1731 | No |
| 68 | NUDT15 |  |  | 4932 | -0.030 | -0.1900 | No |
| 69 | PRKAB1 |  |  | 4945 | -0.031 | -0.1900 | No |
| 70 | MDM2 |  |  | 4954 | -0.031 | -0.1896 | No |
| 71 | TOB1 |  |  | 5047 | -0.035 | -0.1985 | No |
| 72 | CYFIP2 |  |  | 5050 | -0.035 | -0.1972 | No |
| 73 | ELP1 |  |  | 5056 | -0.035 | -0.1963 | No |
| 74 | BTG1 |  |  | 5087 | -0.037 | -0.1981 | No |
| 75 | RALGDS |  |  | 5182 | -0.039 | -0.2070 | No |
| 76 | TP53 |  |  | 5275 | -0.043 | -0.2155 | No |
| 77 | PPP1R15A |  |  | 5321 | -0.045 | -0.2186 | No |
| 78 | DDIT3 |  |  | 5335 | -0.046 | -0.2181 | No |
| 79 | HEXIM1 |  |  | 5341 | -0.046 | -0.2167 | No |
| 80 | BAK1 |  |  | 5425 | -0.049 | -0.2239 | No |
| 81 | FBXW7 |  |  | 5435 | -0.049 | -0.2228 | No |
| 82 | NDRG1 |  |  | 5645 | -0.060 | -0.2438 | No |
| 83 | RRP8 |  |  | 5699 | -0.062 | -0.2472 | No |
| 84 | ZNF365 |  |  | 5731 | -0.063 | -0.2479 | No |
| 85 | FDXR |  |  | 6009 | -0.074 | -0.2760 | No |
| 86 | RCHY1 |  |  | 6024 | -0.076 | -0.2743 | No |
| 87 | PPM1D |  |  | 6025 | -0.076 | -0.2711 | No |
| 88 | RAP2B |  |  | 6059 | -0.077 | -0.2714 | No |
| 89 | APAF1 |  |  | 6128 | -0.081 | -0.2756 | No |
| 90 | MAPKAPK3 |  |  | 6220 | -0.086 | -0.2822 | No |
| 91 | SERTAD3 |  |  | 6224 | -0.086 | -0.2788 | No |
| 92 | FOXO3 |  |  | 6238 | -0.087 | -0.2765 | No |
| 93 | TAX1BP3 |  |  | 6254 | -0.088 | -0.2744 | No |
| 94 | WWP1 |  |  | 6518 | -0.102 | -0.2997 | No |
| 95 | RGS16 |  |  | 6543 | -0.104 | -0.2979 | No |
| 96 | HMOX1 |  |  | 6561 | -0.106 | -0.2952 | No |
| 97 | PTPN14 |  |  | 6704 | -0.114 | -0.3063 | No |
| 98 | CCND3 |  |  | 6881 | -0.128 | -0.3207 | No |
| 99 | TRAF4 |  |  | 6895 | -0.129 | -0.3166 | No |
| 100 | ZMAT3 |  |  | 6960 | -0.134 | -0.3180 | No |
| 101 | DDIT4 |  |  | 6968 | -0.135 | -0.3130 | No |
| 102 | RAD51C |  |  | 6989 | -0.136 | -0.3094 | No |
| 103 | TXNIP |  |  | 7037 | -0.140 | -0.3086 | No |
| 104 | CCNG1 |  |  | 7061 | -0.143 | -0.3051 | No |
| 105 | PLK2 |  |  | 7069 | -0.143 | -0.2997 | No |
| 106 | PHLDA3 |  |  | 7307 | -0.168 | -0.3192 | No |
| 107 | PMM1 |  |  | 7371 | -0.177 | -0.3187 | No |
| 108 | ZFP36L1 |  |  | 7466 | -0.188 | -0.3212 | No |
| 109 | IRAK1 |  |  | 7477 | -0.189 | -0.3141 | No |
| 110 | GADD45A |  |  | 7633 | -0.208 | -0.3226 | Yes |
| 111 | MXD4 |  |  | 7641 | -0.209 | -0.3144 | Yes |
| 112 | DCXR |  |  | 7713 | -0.220 | -0.3129 | Yes |
| 113 | UPP1 |  |  | 7849 | -0.243 | -0.3176 | Yes |
| 114 | NINJ1 |  |  | 7913 | -0.258 | -0.3136 | Yes |
| 115 | JUN |  |  | 7930 | -0.262 | -0.3040 | Yes |
| 116 | FAM162A |  |  | 7983 | -0.272 | -0.2982 | Yes |
| 117 | EI24 |  |  | 8003 | -0.276 | -0.2884 | Yes |
| 118 | TRIAP1 |  |  | 8060 | -0.288 | -0.2822 | Yes |
| 119 | TPRKB |  |  | 8072 | -0.291 | -0.2709 | Yes |
| 120 | PCNA |  |  | 8082 | -0.293 | -0.2592 | Yes |
| 121 | RPS27L |  |  | 8126 | -0.302 | -0.2510 | Yes |
| 122 | SAT1 |  |  | 8162 | -0.314 | -0.2414 | Yes |
| 123 | ISCU |  |  | 8183 | -0.321 | -0.2298 | Yes |
| 124 | TSC22D1 |  |  | 8334 | -0.363 | -0.2310 | Yes |
| 125 | LDHB |  |  | 8372 | -0.379 | -0.2188 | Yes |
| 126 | BAX |  |  | 8426 | -0.398 | -0.2075 | Yes |
| 127 | HRAS |  |  | 8633 | -0.514 | -0.2086 | Yes |
| 128 | CDKN1A |  |  | 8679 | -0.545 | -0.1901 | Yes |
| 129 | RACK1 |  |  | 8814 | -0.664 | -0.1765 | Yes |
| 130 | RPL18 |  |  | 8907 | -0.780 | -0.1532 | Yes |
| 131 | RPL36 |  |  | 8931 | -0.843 | -0.1193 | Yes |
| 132 | HINT1 |  |  | 8961 | -0.923 | -0.0827 | Yes |
| 133 | S100A10 |  |  | 8965 | -0.932 | -0.0427 | Yes |
| 134 | RPS12 |  |  | 8975 | -1.034 | 0.0010 | Yes |
Table: GSEA details [plain text format]

  

Fig 2: HALLMARK\_P53\_PATHWAY: Random ES distribution      
 Gene set null distribution of ES for **HALLMARK\_P53\_PATHWAY**
